# Is the cost of the new home dialysis techniques still advantageous compared to in-center hemodialysis? An Italian single center analysis and comparison with experiences from western countries

**DOI:** 10.3389/fmed.2024.1345506

**Published:** 2024-03-11

**Authors:** Gian Maria Iadarola, Elisa Giorda, Marco Borca, Daniela Morero, Savino Sciascia, Dario Roccatello

**Affiliations:** University Center of Excellence on Nephrological, Rheumatological and Rare Diseases (ERK-net, ERN-Reconnect and RITA-ERN Member) Including Nephrology and Dialysis Unit and Center of Immuno-Rheumatology and Rare Diseases (CMID), Coordinating Center of the Interregional Network for Rare Diseases of Piedmont and Aosta Valley (North-West Italy), San Giovanni Bosco Hub Hospital, ASL Città di Torino and Department of Clinical and Biological Sciences of the University of Turin, Turin, Italy

**Keywords:** home dialysis, home dialysis costs, home dialysis care, dialysis, dialysis patient

## Abstract

**Introduction:**

Potential advantages of home dialysis remained a questionable issue. Three main factors have to be considered: the progressive reduction in the cost of consumables for in-Center hemodialysis (IC-HD), the widespread use of incremental Peritoneal Dialysis (PD), and the renewed interest in home hemodialysis (H-HD) in the pandemic era. Registries data on prevalence of dialysis modalities generally report widespread underemployment of home dialysis despite PD and H-HD could potentially provide clinical benefits, improve quality of life, and contrast the diffusion of new infection among immunocompromised patients.

**Methods:**

We examined the economic impact of home dialysis by comparing the direct and indirect costs of PD (53 patients), H-HD (21 patients) and IC-HD (180 patients) in a single hospital of North-west Italy. In order to achieve comparable weekly costs, the average weekly frequency of dialysis sessions based on the dialysis modality was calculated, the cost of individual sessions per patient per week normalized, and the monthly and yearly costs were derived.

**Results:**

As expected, PD resulted the least expensive procedure (€ 23,314.79 per patient per year), but, notably, H-HD has a lower average cost than IC-HD (€ 35,535.00 vs. € 40,798.98). A cost analysis of the different dialysis procedures confirms the lower cost of PD, especially continuous ambulatory PD, compared to any extracorporeal technique.

**Discussion:**

Among the hemodialysis techniques, home bicarbonate HD showed the lowest costs, while the weekly cost of Frequent Home Hemodialysis was found to be comparable to In-Center Bicarbonate Hemodialysis.

## Introduction

According to recent estimates, Italy has 53,000 patients on dialysis, 54,000 hospitalizations for renal failure and 10,000 new patients/year starting dialysis, resulting in an estimated €2.4 billion in both direct and indirect costs for the Italian national health system (NHS) (1). In recent estimates, the Italian public health care expenditure made up 6.6% of the gross domestic product, i.e., €113 out of €1,721 billion (2). The overall cost of dialysis in Italy has shown to be approximately € 2.0 billion, equal to 1.79% of the total expenditure of the national health service (2). On average a dialysis patient consumes 22.7 more resources than the average Italian citizen (3). The financial impact of the different techniques of renal replacement therapy has an obvious interest for the sustainability of the health system over time. Three main factors must be considered (4, 5): the progressive reduction in the cost of consumables for IC-HD, the widespread use of incremental PD, and renewed interest of H-HD in the pandemic era. There is a general consensus that PD and H-HD offer consistent advantages over IC-HD in terms of social and occupational indicators (6, 7), clinical benefits, and financial burden. Nevertheless, IC-HD is far more widespread than home dialysis (approx. 89.5% vs. 10.5% in Italy) (8).

We report the results of an observational, single-center study on the financial aspects associated with the various dialysis modalities in Piedmont, North-west Italy.

The main interest of this evaluation was to identify the cost of the specific treatment modalities, including In-Center Bicarbonate Hemodialysis (IC-BHD), In-Center Hemodiafiltration (IC-HDF), Continuous Ambulatory Peritoneal Dialysis (CAPD), Automated Peritoneal Dialysis (APD), Frequent Home Hemodialysis (FH-HD), Home Bicarbonate Hemodialysis (H-BHD).

## Methods

This study was conducted at the Nephrology and Dialysis Unit of the San Giovanni Bosco Hub Hospital and University of Turin, Italy, the main facility for dialysis in the Northern part of the metropolitan area of Turin from January 1, 2019 to December 31, 2019.

Costs of 52,673 consecutive dialysis treatments (27,588 IC-HD; 20,262 PD; 4,823 H-HD) delivered in a year were analyzed.

The cost calculation methodology, definitions of direct and indirect costs, and differences across the type of treatment are detailed in the Supplementary materials. The main search characteristics of the scoping review strategies are also provided.

The study was conducted from the perspective of a Hub-Hospital as part of the Italian National Health Service.

## Results

Direct and indirect costs of IC-BHD, IC-HDF, IC-AFB, CAPD, APD, FH-HD, H-BHD in euro per patient/week measured at the Unit of Nephrology and Dialysis of the S. Giovanni Bosco Hub Hospital between 1 January and 31 December 2019, are shown in Table 1.

**Table 1:** Direct and indirect costs of IC-BHD, IC-HDF, IC-AFB, CAPD, APD, FH-HD, H-BHD in euro per patient/week measured at the Unit of Nephrology and Dialysis of the S. Giovanni Bosco Hub Hospital and University of Turin between January 1st and December 31st, 2019.

	IC-BHD	IC-HDF	IC-AFB	CAPD	APD	H-FHD	H-BHD
Pts n. (weekly mean)	103	66	11	40	13	16	5
Direct costs (€)							
Dialysis consumables	75.40	100.12	139.13	231.00	381.38	445.00	180.95
Monitor expenses	39.75	39.75	39.75	0.00	0.00	93.10	65.17
Bed weight scale	5.55	5.55	5.55	2.08	2.01	1.57	1.10
Drugs	72.00	72.00	72.00	18.57	17.90	77.85	54.50
Storage	11.86	11.86	11.86	0.00	0.00	0.00	0.00
Water purification	6.97	6.97	6.97	0.00	0.00	0.00	0.00
Medical staff	45.39	45.39	45.39	23.46	22.62	40.23	28.16
Internal nursing staff	168.75	168.75	168.75	79.83	76.98	77.79	54.45
External nursing staff	109.28	109.28	109.28	0.00	0.00	0.00	0.00
Total	534.95	559.67	598.68	354.94	500.89	775.41	412.24
Indirect costs	IC-BHD	IC-HDF	IC-AFB	CAPD	APD	H-FHD	H-BHD
Transportation	141.32	141.32	141.32	2.50	2.41	0.00	0.00
Dialysis Center maintenance	90.34	90.34	141.32	53.15	51.25	39.87	27.91
Total	231.66	231.66	282.65	55.65	53.67	39.87	27.91
Total direct + Indirect costs	766.61	791.34	881.33	410.60	554.56	775.41	412.24

The cost analysis of IC-BHD, IC-HDF and IC-AFB showed average costs per patient, per year as €39,973.38, €41,262.52, and €45,954.92, respectively. The average costs measured for CAPD and APD were €21,409.64, and €28,916.17 per patient, per year, respectively. Regarding H-HD, the average costs per patient, per year of H-FHD and H-BHD were €40,432.09 and €21,495.21, respectively. Costs are summarized in Table 2.

**Table 2:** Cost of IC-BHD, IC-HDF, IC-AFB, CAPD, APD, FH-HD, H-BHD in euro per patient/week, month and year, measured at the Unit of Nephrology and Dialysis of the S. Giovanni Bosco Hub Hospital and University of Turin between January 1st and December 31st, 2019.

	IC-BHD	IC-HDF	IC-AFB	CAPD	APD	H-FHD	H-BHD
Week	766.61	791.34	881.33	410.60	554.56	775.41	412.24
Month	3,331.11	3,438.54	3,829.57	1,784.13	2,409.681	3,369.341	1,791.268
Year	39,973.38	41,262.52	45,954.92	21,409.64	28,916.17	40,432.09	21,495.21

CAPD is the least expensive dialysis modality, while H-BHD is by far the least expensive of the extracorporeal treatments, even when compared to a PD technique such as APD. H-FHD is competitive compared to IC-BHD, while IC-HDF and IC-AFB are more expensive.

**Figure 1:**
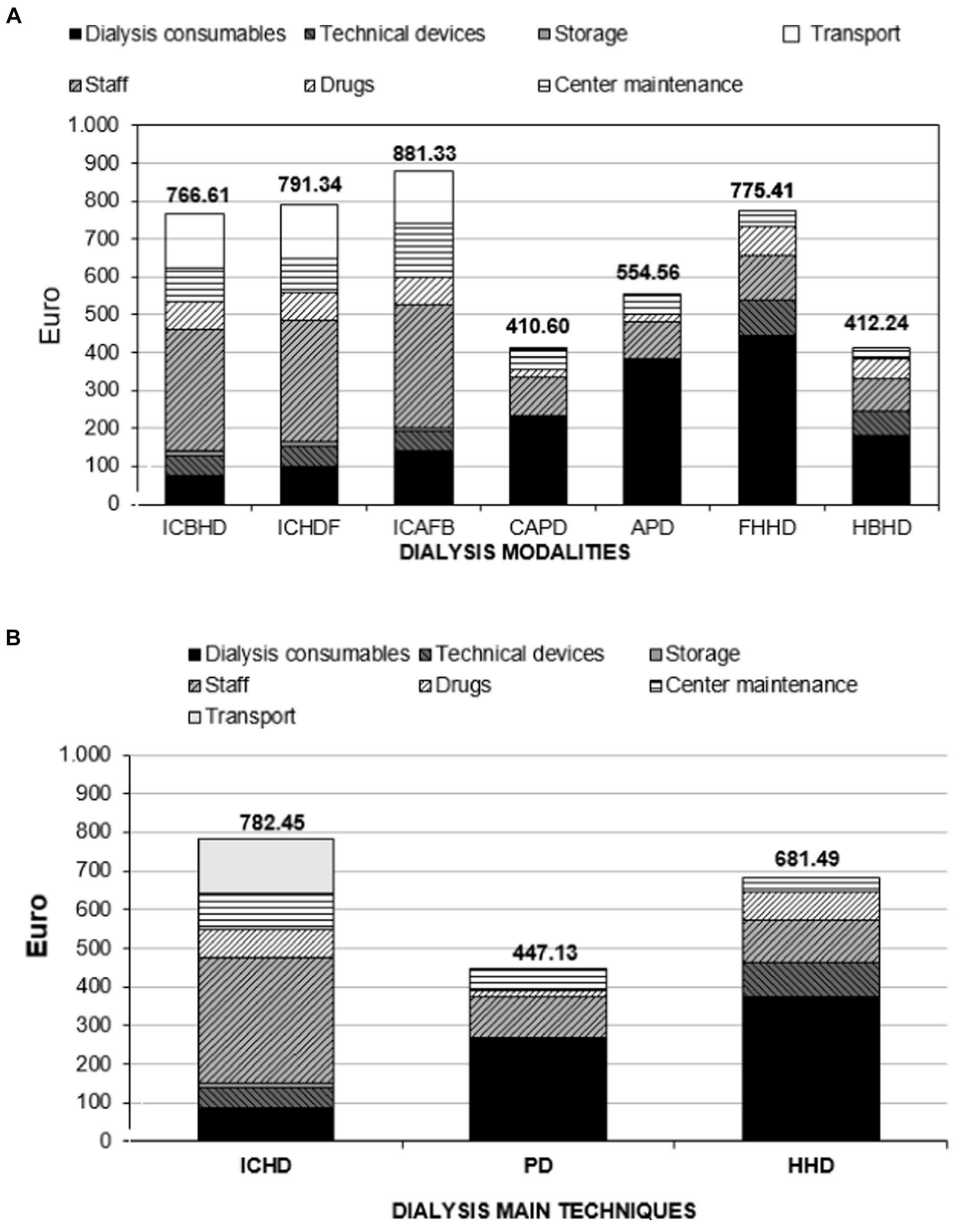
**(A)** Direct and indirect dialysis costs in Euro. Weekly weight of each budget item per dialysis modality. Data from the Nephrology and Dialysis Unit of the S. Giovanni Bosco Hub Hospital, Turin, Italy, 2019. **(B)** Direct and indirect dialysis costs in Euro. Weekly weight of each budget item per main dialysis modality. Data from the Nephrology and Dialysis Unit of the S. Giovanni Bosco Hub Hospital, Turin, Italy, 2019.

For the main techniques, the total direct and indirect costs of IC-HD compared to PD and H-HD are shown in Table 3. In comparison to home dialysis, IC-HD has higher average dialysis session costs per week, per month, and per year. Peritoneal dialysis shows the lowest overall cost (per year: IC-HD €40,798.98; PD €23,314.79; H-HD €35,535.00) as seen in Figure 1B.

**Table 3:** Total direct and indirect costs of IC-HD compared to PD and H-HD in euro, measured at the Unit of Nephrology and Dialysis of the S. Giovanni Bosco Hub Hospital and University of Turin between January 1st and December 31st, 2019.

	In center-hemodialysis	Peritoneal dialysis	Home hemodialysis
Week	782.45	447.13	681,49
Month	3,399.91	1,942.90	2,961.25
Year	40,798.98	23,314.79	35,535.00

Healthcare personnel, facility maintenance and transportation costs were found to be the most expensive items of all the modalities of IC-HD.

Dialysis consumables represent the greatest single expense for H-FHD due to the higher frequency of sessions. Furthermore, H-HD costs are affected by other budget items, such as monitor installation, waste disposal and periodic supply of devices, whereas the mini-osmosis system and periodic microbiological water monitoring also have an impact on H-BHD costs. The costs related to H-BHD are very low because this type of treatment combines a reduction of the costs of consumables along with the low staff costs and the absence of transportation costs.

The daily costs of peritoneal dialysis include expenses for cyclers, home consumable supplies, and medical professional assistance. Each budget item included in the overall weekly cost for each dialysis modality is shown in Figure 1A. While the cost of consumables has become very low, the expense for thrice-weekly IC-HD remains more burdensome than H-HD due to the high costs of the healthcare staff, transportation, and Center maintenance.

To compare our results with the international panorama, with scoping review approach, data as summarized in Table 4. We retrieved a remarkable difference across countries, also due to heterogeneity in study design, considered variables and time of the analysis.

**Table 4:** Overview of HD, home HD, and PD costs in Europe.

Country	Year	N	HD		Home HD		PD				References
Austria	2001–2008	-	43.600–40.000€		-		25.900–15.300€				(9)
Belgium	2009	-	61.708 €		48.354–40.256€		48.226–38.176€ (CAPD)		42.486€ (APD)		(10)
Denmark	1998	-	392.000–328.000 DKK (52.514–43.940€)		-		CAPD		APD		(11)
							291.000–251.000 DKK (38.983–33.625€)		325.000–296.000 DKK (43.538–39.653€)		
France	2005	-	81.500–59.500€		49.900 €		50.000€ (CAPD)		49.700€ (APD)		(12)
France	2007	60.900	89.000 €		-		64.000 €				(13)
Finland	1991–1996	214	54.490–54.140$		-		49.299–45.262$ (CAPD)		-		(14)
Finland	2003–2004	29	77.126 €		-		55.743 €				(15)
Greece	2008	707	36.247 €		-		30.719 €				(16)
Netherlands	2012–2014	4.209	92.616 €		87.051 €		77.566€ (CAPD)		89.932€ (APD)		(17)
			RRT 71.734€	No RRT 20.882€	RRT 72.834€	No RRT 14.217€	RRT 61.025€	No RRT 16.541€	RRT 74.215€	No RRT 15.717€	
Spain	1994	-	55.076–49.767$		-		31.201$ (CAPD)		42.519$ (APD)		(18)
Spain	2010	6.231	Direct 35.313-	Indirect 8.929€	Direct 46.344-	Indirect 8.929€	Direct CAPD 24.028€	Indirect CAPD 7.429€	Direct APD 34.045€	Indirect APD 7.429€	(19)
			31.646 €		42.677 €						
Spain	2011	-	21.595 €		-		25.664 €				(20)
Sweden	2010	561	87.600 €		-		58.600 €				(21)
UK	2005	41.776	35.023–32.669£		20.764£		15.570£ (CAPD)		21.655£ (APD)		(22)
Turkey	2000	-	22.759$		-		22.350$ (CAPD)		-		(23)

HD, Hemodialysis; PD, Peritoneal dialysis; CAPD, Continuous ambulatory PD; APD, Automated PD; RRT, Renal replacement therapy; No RRT, Costs due to hospital or ambulatory care not related to HD itself.

## Discussion

The present study shows that home dialysis has lower overall costs than in-Center hemodialysis despite the burden of consumables for the higher frequency of treatment.

Compared to other studies that were carried out without distinction among home hemodialysis treatment techniques, it is worth noticing that H-BHD performed every other day further reduces the overall average costs of the home hemodialysis system and may be even competitive with APD. PD and particularly CAPD have shown the most cost-effective profile (4, 24, 25).

These data could possibly be affected in future by a change in the cost of consumables (as the costs of consumables for IC-HD have been progressively decreased in recent years) and the costs of staff salaries. One could speculate that an increase in wages could result in an additional financial advantage for home dialysis due to favorable ratio between patients and personnel (26). Choosing among the different modalities of dialysis is a multifaced aspect and cost effectiveness is only one variable to consider. Nevertheless, the potential benefits of home dialysis rely on a reduction of both direct and indirect costs (e.g., reduced need for technical positions when compared to hospital hemodialysis and consequently reduced costs for structures and personnel). An obvious limitation of this study is that data have been derived from a single-Center observation. Additionally, due to the Center’s policy, home hemodialysis programs are defined as H-FHD programs with portable monitors, low flow and high saturation of dialysate (supplied in preformed bags), frequency > 4 sessions/week, and H-BHD programs with self-produced dialysate, mini-osmosis and dialysis every other day. Also, no specific analysis has been performed to quantify the cost of the development of self-training and manpower development. Finally, no quality-adjusted life year scoring or patients’ reported outcomes have computed in our analysis, limiting the strength our results in terms of patients’ experience.

The following issues were not assessed in this analysis: costs of hospitalizations, costs related to vascular access and management of vascular access complications for hemodialysis patients, costs related to the surgical insertion of the peritoneal dialysis catheter and possible complications for peritoneal dialysis, and costs associated with PD and H-HD training. Social costs, such as the time lost by patients and caregivers from working, were also not assessed.

Some further aspects are worth mentioning. Although the difference between H-FHD and IC-BHD in our cost analysis is not obvious, cost-utility analyses demonstrate that H-FHD is still financially competitive compared to IC-HD when measuring the results in terms of QALYS (4, 24, 25).

On the other hand, H-FHD is not necessarily synonymous with H-HD, and portable low-flow and high-saturation dialysate hemodialysis systems should not be regarded as such, although their availability has proven to be highly valuable for the modern development of home hemodialysis. While the multiple clinical advantages of frequent hemodialysis are well known, not all patients have a strict need for it. Furthermore, patients of large body size and with no substantial need for transportability of dialysis are always willing to undergo frequent weekly treatments to achieve adequate purification targets with low-flow and high-saturation dialysate treatments. This is especially true when H-BHD on alternate days can carry out the task just as well. Indeed, it is necessary to seek a balance between clinical, logistical, and quality of life aspects, as well as socio-working context. A patient-tailored H-HD system that incorporates several kinds of home treatment (CAPD, APD, H-FHD, H-BHD), and that is used in relation to real clinical needs, logistics, and quality of life, can maintain some economic advantage over IC-HD.

When comparing our results to the international panorama, a significant degree in heterogeneity (as summarized in Table 4) exists. Among others, Vanholder et al. (27) performed an analysis using data collected from various units in Belgium, Germany, The Netherlands, England, and France. This analysis aimed to determine the annual cost per HD patient in these countries. The findings revealed that the estimated costs per HD patient were roughly €66,212, €44,575, €65,739, €29,322, and €53,758 for Belgium, Germany, The Netherlands, England, and France, respectively (9–23, 27–36). The anticipated yearly cost in Greece, encompassing all treatment-related expenses, amounts was also estimated to €39,258.50. In the United States, the average yearly cost per dialysis patient is $87,638, as reported in the years 2012 to 2019 (27–34). Conversely, in Canada, the corresponding annual cost is $51,252 during the same time (27–34). At the same time, one should acknowledge that a vast heterogeneity exists in the methodologies used across different studies and in the considered time frame. For example, the cost estimates for dialysis in the United States and the Netherlands encompass the expenses associated with drugs, specifically erythropoietin, vitamin D, and iron. The cost estimates for France and Canada did not incorporate the expenses related to erythropoietin (9, 27–35). Besides, costs can vary significantly from one country to another and may depend on a variety of additional factors, including the healthcare system, public or private healthcare providers, and individual insurance coverage.

## Conclusion

Our data analysis confirms the low cost of CAPD and PD in general. H-BHD is so advantageous that it has lower costs not only compared to all other hemodialysis modalities, but is competitive to APD, while the weekly cost of H-FHD is comparable to that of IC-BHD.

A home hemodialysis system that offers bicarbonate hemodialysis on alternate days in addition to frequent hemodialysis seems to be more cost-effective overall than conventional in-Center hemodialysis, including IC-BHD, IC-HDF, and IC-AFB.

Taken together, these data, especially in the uncertainty of possible new pandemic waves, can be a reason for reflection and a possible change of strategy of renal replacement therapy.

## Data availability statement

The raw data supporting the conclusions of this article will be made available by the authors, without undue reservation.

## Ethics statement

The requirement of ethical approval was waived by CET Interaziendale AOU Città della Salute e della Scienza di Torino for the studies involving humans because observational audit reviewing costs. The studies were conducted in accordance with the local legislation and institutional requirements. The ethics committee/institutional review board also waived the requirement of written informed consent for participation from the participants or the participants’ legal guardians/next of kin because observational audit reviewing costs.

## Author contributions

GI: Conceptualization, Data curation, Formal analysis, Investigation, Methodology, Supervision, Writing – original draft, Writing – review & editing. EG: Data curation, Formal analysis, Investigation, Writing – review & editing. MB: Data curation, Formal analysis, Methodology, Writing – review & editing. DM: Data curation, Formal analysis, Investigation, Methodology, Writing – review & editing. SS: Formal analysis, Methodology, Validation, Writing – review & editing. DR: Conceptualization, Data curation, Formal analysis, Investigation, Methodology, Validation, Writing – original draft, Writing – review & editing.

## Figures and Tables

**Table:** 

CKD	Chronic kidney disease
RRT	Renal replacement therapy
IC-HD	In-center hemodialysis
PD	Peritoneal dialysis
H-HD	Home hemodialysis
IC-BHD	In-Center Bicarbonate Hemodialysis
IC-HDF	In-Center Hemodiafiltration (also online)
IC-AFB	In-Center Acetate Free Biofiltration
CAPD	Continuous ambulatory peritoneal dialysis
APD	Automated peritoneal dialysis
FH-HD	Frequent home hemodialysis
H-BHD	Home bicarbonate hemodialysis
QoL	Quality of life
ESRD	End stage renal disease
NHS	National health system
GDP	Gross domestic product
€	Euro
QALYS	Quality adjusted life years

## References

[1] Cicchetti A Ruggeri M Codella P Ridolfi A. I costi socio-sanitari dell’insufficienza renale cronica. Farmacoeconomia e percorsi terapeutici. (2011) 12:21–8.

[2] Corte dei Conti, Sezione Autonomie. (2019). “Referto Al Parlamento Sulla Gestione Finanziaria Dei Servizi Sanitari Regionali” Esercizio 2017 Deliberazione N. Available at: https://www.corteconti.it/Download?id=03d77748-7297-4130-95aa-47ec6d8ee045

[3] ISTAT. (2019). Statistiche demografiche ISTAT, Istituto Nazionale di Statistica. Available at: http://demo.istat.it/

[4] Walker R Marshall MR Morton RL McFarlane P Howard K. The cost‐effectiveness of contemporary home haemodialysis modalities compared with facility haemodialysis: a systematic review of full economic evaluations. Nephrology (Carlton). (2014) 19:459–70. doi: 10.1111/nep.12269, PMID: 24750559

[5] Mowatt G Vale L Perez J Wyness L Fraser C MacLeod A. Systematic review of the effectiveness and cost-effectiveness, and economic evaluation, of home versus hospital or satellite unit haemodialysis for people with end-stage renal failure. Health Technol Assess. (2003) 7:1–174. doi: 10.3310/hta7020, PMID: 12773260

[6] Walker RC Howard K Tong A Palmer SC Marshall MR Morton RL. The economic considerations of patients and caregivers in choice of dialysis modality. Hemodial Int. (2016) 20:634–42. doi: 10.1111/hdi.12424, PMID: 27196634 PMC5324572

[7] Liu FX Treharne C Culleton B Crowe L Arici M. The financial impact of increasing home-based high dose haemodialysis and peritoneal dialysis. BMC Nephrol. (2014) 15:161. doi: 10.1186/1471-2369-15-161, PMID: 25278356 PMC4194367

[8] Società Italiana di Nefrologia. (2015). Registro Italiano di Dialisi e Trapianto. Report 2015. Available at: https://ridt.sinitaly.org/2017/10/09/report-2015/

[9] Johnson DW Vincent K Blizzard S. Cost savings from peritoneal dialysis therapy time extension using Icodextrin. Adv Perit Dial. (2003) 19:81–5. PMID: 14763039

[10] Haller M Gutjahr G Kramar R Harnoncourt F Oberbauer R. Cost-effectiveness analysis of renal replacement therapy in Austria. Nephrol Dial Transplant. (2011) 26:2988–95. doi: 10.1093/ndt/gfq780, PMID: 21310740

[11] KCE. (2010). Organisation et financement de la dialyse chronique en Belgique. Available at: https://kce.fgov.be/fr/publications/tous-les-rapports/organisation-et-financement-de-la-dialyse-chronique-en-belgique (Accessed October 26, 2023).

[12] Maschoreck TR Sørensen MC Andresen M Høgsberg IM Rasmussen P Søgaard J. Omkostningsanalyse af dialysebehandlingen på Odense Universitetshospital og Sønderborg Sygehus [cost analysis of dialysis treatment at the Odense University Hospital and the Sønderborg hospital]. Ugeskr Laeger. (1998) 160:7418–24. PMID: 9889655

[13] Benain JP Faller B Briat C Jacquelinet C Brami M Aoustin M. Coût de la prise en charge de la dialyse en France [Cost of dialysis in France]. Nephrol Ther. (2007) 3:96–106. doi: 10.1016/j.nephro.2007.03.001, PMID: 17540311

[14] Blotière PO Tuppin P Weill A Ricordeau P Allemand H. Coût de la prise en charge de l’IRCT en France en 2007 et impact potentiel d’une augmentation du recours à la dialyse péritonéale et à la greffe [The cost of dialysis and kidney transplantation in France in 2007, impact of an increase of peritoneal dialysis and transplantation]. Nephrol Ther. (2010) 6:240–7. doi: 10.1016/j.nephro.2010.04.005 20554257

[15] Salonen T Reina T Oksa H Sintonen H Pasternack A. Cost analysis of renal replacement therapies in Finland. Am J Kidney Dis. (2003) 42:1228–38. doi: 10.1053/j.ajkd.2003.08.024, PMID: 14655195

[16] Hallinen T Soini EJ Martikainen JA Ikäheimo R Ryynänen OP. Costs and quality of life effects of the first year of renal replacement therapy in one Finnish treatment Centre. J Med Econ. (2009) 12:136–40. doi: 10.3111/13696990903119530, PMID: 19566482

[17] Kontodimopoulos N Niakas D. An estimate of lifelong costs and QALYs in renal replacement therapy based on patients’ life expectancy. Health Policy. (2008) 86:85–96. doi: 10.1016/j.healthpol.2007.10.002, PMID: 17996975

[18] Mohnen SM van Oosten MJM Los J Leegte MJH Jager KJ Hemmelder MH. Healthcare costs of patients on different renal replacement modalities – analysis of Dutch health insurance claims data. PLoS One. (2019) 14:e0220800. doi: 10.1371/journal.pone.0220800, PMID: 31415578 PMC6695145

[19] Rodríguez-Carmona A Perez Fontán M Bouza P García Falcón T Valdés F. The economic cost of dialysis: a comparison between peritoneal dialysis and in-center hemodialysis in a Spanish unit. Adv Perit Dial. (1996) 12:93–6. PMID: 8865880

[20] Villa G Rodríguez-Carmona A Fernández-Ortiz L Cuervo J Rebollo P Otero A. Cost analysis of the Spanish renal replacement therapy programme. Nephrol Dial Transplant. (2011) 26:3709–14. doi: 10.1093/ndt/gfr088, PMID: 21427072

[21] Lamas Barreiro JM Alonso Suárez M Saavedra Alonso JA Gándara MA. Costs and added value of haemodialysis and peritoneal dialysis outsourcing agreements. Nefrologia. (2011) 31:656–63. doi: 10.3265/Nefrologia.pre2011.Oct.11032, PMID: 22130280

[22] Eriksson JK Neovius M Jacobson SH Elinder CG Hylander B. Healthcare costs in chronic kidney disease and renal replacement therapy: a population-based cohort study in Sweden. BMJ Open. (2016) 6:e012062. doi: 10.1136/bmjopen-2016-012062, PMID: 27855091 PMC5073563

[23] Baboolal K McEwan P Sondhi S Spiewanowski P Wechowski J Wilson K. The cost of renal dialysis in a UK setting--a multicentre study. Nephrol Dial Transplant. (2008) 23:1982–9. doi: 10.1093/ndt/gfm870, PMID: 18174268

[24] Beby AT Cornelis T Zinck R Liu FX. Cost-effectiveness of high dose Hemodialysis in comparison to conventional in-Center Hemodialysis in the Netherlands. Adv Ther. (2016) 33:2032–48. doi: 10.1007/s12325-016-0408-4, PMID: 27664108

[25] Weinhandl ED. Economic impact of home Hemodialysis. Adv Chronic Kidney Dis. (2021) 28:136–42. doi: 10.1053/j.ackd.2021.06.010, PMID: 34717859

[26] Manns B Agar JWM Biyani M Blake PG Cass A Culleton B. Can economic incentives increase the use of home dialysis? Nephrol Dial Transplant. (2019) 34:731–41. doi: 10.1093/ndt/gfy223 30010852

[27] Vanholder R Davenport A Hannedouche T Kooman J Kribben A Lameire N. Reimbursement of dialysis: a comparison of seven countries. J Am Soc Nephrol. (2012) 23:1–8. doi: 10.1681/ASN.2011111094 22677554

[28] Francisco A Kim J Anker SD Belozeroff V Canaud B Chazot C. An epidemiological study of hemodialysis patients based on the European Fresenius medical care Hemodialysis network: results of the ARO study. Nephron Clin Pract. (2011) 118:c143–54. doi: 10.1159/000319936, PMID: 21150222

[29] Saran R Robinson B Abbott KC Agodoa LYC Albertus P Ayanian J. US renal data system 2016 annual data report: Epidemiology of kidney disease in the United States. Am J Kidney Dis. 69:A7–8. doi: 10.1053/j.ajkd.2016.12.004 PMC6605045 28236831

[30] Lee H Manns B Taub K Ghali WA Dean S Johnson D. Cost analysis of ongoing care of patients with end-stage renal disease: the impact of dialysis modality and dialysis access. Am J Kidney Dis. (2002) 40:611–22. doi: 10.1053/ajkd.2002.34924, PMID: 12200814

[31] Murphy SW Foley RN Barrett BJ Kent GM Morgan J Barré P. Comparative hospitalization of hemodialysis and peritoneal dialysis patients in Canada. Kidney Int. (2000) 57:2557–63. doi: 10.1046/j.1523-1755.2000.00115.x, PMID: 10844625

[32] Blake P Just P. Economics of dialysis In: Horl WH Koch KM Lindsay RM Ronco C Winchester JF, editors. Replacement of renal function by Dialysis. 5th ed. Dordrecht: Kluwer (2004)

[33] Weijnen TJ van Hamersvelt HW Just PM. Economic impact of extended time on peritoneal dialysis as a result of using polyglucose: the application of a Markov chain model to forecast changes in the development of the ESRD programme over time. Nephrol Dial Transplant. (2003) 18:390–6. doi: 10.1093/ndt/18.2.390, PMID: 12543897

[34] Huang HC Wang JY Chang CC Chiu PF Chiang MC Yang Y. Nonclinical factors associated with treatment with peritoneal dialysis in ESRD patients in Taiwan. Perit Dial Int. (2010) 30:638–43. doi: 10.3747/pdi.2009.00112, PMID: 20448240

[35] Durand PY Verger C. The state of peritoneal dialysis in France. Perit Dial Int. (2006) 26:654–7. doi: 10.1177/089686080602600608, PMID: 17047231

[36] Erek E Sever MS Akoglu E Sariyar M Bozfakioglu S Apaydin S. Cost of renal replacement therapy in Turkey. Nephrology (Carlton). (2004) 9:33–8. doi: 10.1111/j.1440-1797.2003.00218.x, PMID: 14996307

