# Supplementary material for: Is the cost of the new home dialysis techniques still advantageous compared to in-center hemodialysis? An Italian single center analysis and comparison with experiences from western countries
Source: Front Med (Lausanne). 2024 Mar 11;11:1345506. doi: 10.3389/fmed.2024.1345506 (PMC10961330; doi:10.3389/fmed.2024.1345506)
Supplement: Supplementary file 1 [file Data_Sheet_1.docx]

**Supplementary material**

The cost calculation methodology, the definitions of direct/indirect costs, and costs differences across the types of treatments are detailed as follows.

COST CALCULATION METHODOLOGY

***Definitions***

*Direct costs.* Costs directly related to dialysis treatment (including dialysis consumables, healthcare staff, medication provided by the centralised pharmacy service, supplies and storage costs of dialysis consumables). Direct costs of dialysis consumables were derived from the prices of the supply’s contracts divided by the number of procedures delivered/year. Healthcare staff costs were extrapolated from the gross salaries of each staff member (physicians, nurses, other healthcare workers) and they were calculated based on the amount of time that was dedicated to each dialysis program. The consumables storage warehouse for IC-HD is outsourced to a private management firm that also provides the daily supply of consumables to the dialysis centre.

The costs of the nursing staff (beside nursing service directly employed by the San Giovanni Bosco Hub Hospital) included those outsourced to a private service. The privately outsourced nursing staff manages approximately half of IC-HD patients. When referring to the home dialysis, the remote assistance and management was related to the APD only. The nursing staff involved in the activities was directly employed by the San Giovanni Bosco Hub Hospital. At the time of study, 13 patients were receiving APD. Due to these motivations, the remote assistance and management was conducted as iso-resource with no additional costs.

*Indirect costs*. Maintenance costs for dialysis or other costs that enable treatment but are not directly related to the procedure (such as transportation costs and general administrative costs as well as costs related to the dialysis facility's maintenance, such as those related to power and water supply, heating, and cleaning supplies).

The all-inclusive maintenance costs of the dialysis facility and the costs of administrative staff were computed as provided by the hospital's economic and financial management office and calculated for the study period. The costs of transportation were obtained on the basis of the number of transports that were provided to and from the dialysis Centre in 2019 subdivided across the dialysis modalities.

The average cost of consumables required for a single dialysis session was obtained dividing the total costs of materials as indicated by the supply contracts by the number of dialysis sessions budgeted in the supply contracts for each dialysis modality.

The yearly cost of each expenditure item was divided by the number of dialysis sessions completed during the analysis period to get the average cost per single session for dialysis monitors and balance beds, warehousing costs, staff costs, prescription costs, and maintenance costs. For peritoneal dialysis (CAPD and APD), as the contract for the provision of consumables stipulates a flat-rate cost per day regardless of the amount and kind of fluids used, retaining the distinction between CAPD and APD, the cost of the entire treatment day was taken into account.

**Identifying the average cost of the specific treatment modality.** The calculation of the weekly cost for each patient was normalised by multiplying the cost of each dialysis session by the average frequency of each type of treatment (IC-BHD, IC-HDF, IC-AFB 3 sessions/week**;** CAPD 7 days of treatment**/**week, APD 6.75 days of treatment/week; H-FHD 5 treatments/week, H-BHD 3.5 treatments/week).

***Estimating the average cost of the treatment for main techniques.*** *“*Per session cost” was normalized for the mean value of procedures delivered per week, derived from the actual frequency of each patient's treatment programmes: IC-HD 3 sessions per week, PD 6.94 sessions per week, H-HD 4.63 sessions per week.

The weighted average cost for each patient, per week, month and per year was obtained accordingly.

**Literature Search**

The following databases were consulted: PubMed, SCOPUS and WoS using controlled and free terms (dialysis AND COSTS). Articles reporting on costs across the types of treatments in different areas were summarised in Table 4.

It was out of the scope of this study to perform a systematic review of the literature.

Supplementary Tables

|  | **CENSIS - 2009**  **(ITALY)** | **CENSIS - 2009 (PIEDMONT)** | **CICCHETTI – 2010 (ITALY)** |
| --- | --- | --- | --- |
| **IC-HD** | **715.87** | **810.23** | **975.00** |
| **PD** | **551.74** | **582.50** | **574.00** |
| **H-HD** | **-** |  | **-** |

*Tab. 1S Average cost in euro of dialysis treatments (direct health and non-health costs), excluding social costs/non-health indirect costs, in Italy per patient per week, according to dialysis modality (IC-HD 3 dialysis / week; PD 7 dialysis/week).*

|  | **IC-HD** | **PD** | **H-HD** |
| --- | --- | --- | --- |
| **Belgium** | 60,234.76 | 36,897.54 | 39,145.1 |
| **Germany** | 42,366.61 | 40,343.8 | 25,285.11 |
| **The Netherlands** | 62,482.33 | 42,179.32 | 46,674.45 |
| **United Kingdom** | 27,869.81 | 20,864.9 | 27,869.81 |
| **France** | 51,094.66 | 30,772.92 | 30,566.89 |

*Tab. 2S Estimate of the costs of dialysis in Europe, based on reimbursements. Conversion from U.S. dollars to euro at the average exchange value of 2012, mod. from Vanholder,* [Nephrol Dial Transplant. 2016 Aug;31(8):1251-61.]
